# A High-Precision Method of Stiffness Axes Identification for Axisymmetric Resonator Gyroscopes

**DOI:** 10.3390/mi13101793

**Published:** 2022-10-21

**Authors:** Junhao Xiong, Kaiyong Yang, Tao Xia, Jingyu Li, Yonglei Jia, Yunfeng Tao, Yao Pan, Hui Luo

**Affiliations:** College of Advanced Interdisciplinary Studies, National University of Defense Technology, Changsha 410073, China

**Keywords:** Coriolis vibratory gyroscopes, stiffness axes, frequency split, force-to-rebalance mode

## Abstract

Axisymmetric resonators are key elements of Coriolis vibratory gyroscopes (CVGs). The performance of a CVG is closely related to the stiffness and damping symmetry of its resonator. The stiffness symmetry of a resonator can be effectively improved by electrostatic tuning or mechanical trimming, both of which need an accurate knowledge of the azimuth angles of the two stiffness axes of the resonator. Considering that the motion of a non-ideal axisymmetric resonator can be decomposed as two principal oscillations with two different natural frequencies along two orthogonal stiffness axes, this paper introduces a novel high-precision method of stiffness axes identification. The method is based on measurements of the phase difference between the signals detected at two orthogonal sensing electrodes when an axisymmetric resonator is released from all the control forces of the force-to-rebalance mode and from different initial pattern angles. Except for simplicity, our method works with the eight-electrodes configuration, in no need of additional electrodes or detectors. Furthermore, the method is insensitive to the variation of natural frequencies and operates properly in the cases of either large or small frequency splits. The introduced method is tested on a resonator gyroscope, and two stiffness axes azimuth angles are obtained with a resolution better than 0.1°. A comparison of the experimental results and theoretical model simulations confirmed the validity of our method.

## 1. Introduction

The family of Coriolis vibratory gyroscopes (CVGs), currently one of the fastest developing types of gyroscope technologies, can be implemented in a wide range of rotation sensing applications, from low-cost consumer markets to high-end military navigations [1,2,3,4,5,6,7]. They can be divided into two broad categories based on the symmetry of its mechanical element: mode-degenerate gyroscopes and mode-nondegenerate gyroscopes [4,6,8]. As a typical kind of mode-degenerate gyroscope, axisymmetric resonator gyroscopes are the most fascinating for their ability to be operated in the whole-angle mode, which provides a fundamentally unlimited dynamical range and a stable scale factor [4,9].

To develop a high-precision CVG, the two most important characteristics of the resonator are a high-quality factor and the high symmetry of the physical parameters. A high-quality factor is a key feature favored by all kinds of resonators, such as optical resonators [10,11,12], nuclear magnetic resonators [13], and mechanical resonators [6]. For all those resonators, a higher-quality factor corresponds to less energy dissipation, and therefore, lower power consumption is needed and less drive force disturbance is included.

An ideal axisymmetric resonator has perfect stiffness symmetry and no damping. As a result of the imperfections of a real axisymmetric resonator, there is both damping and stiffness asymmetry, which are the two main error sources degrading the performance of the gyroscope [8,14,15,16,17]. Usually, little can be done to reduce the damping asymmetry effectively with repeatability. Stiffness asymmetry leads to a formation of two normal oscillation modes with specific oscillation directions (stiffness axes) and associated natural frequencies. The angle between the two stiffness axes is theoretically determined to be $45^{{}^{\circ}}$ exactly [1,2,3]. The arbitrary motion of the resonator can be regarded as a superposition of the two principal oscillations [1,2,3]. Stiffness asymmetry can be effectively diminished through both mechanical correction and electrostatic correction [18,19,20,21,22]. To perform either mechanical or electrostatic correction accurately and effectively, it is critical to locate the exact stiffness axes orientations of the resonator.

The conventional approach to obtain the stiffness axes of an axisymmetric resonator is through frequency-sweeping tests or frequency responses analyses [23,24,25,26,27]. Most typically, Ding et al. introduced two direct and simple methods by studying the critical points in the magnitude-frequency responses of the two-dimensional transfer function and by analyzing the peak and the valley values of the beat signals at the readout electrodes, respectively, to obtain the stiffness axes based on the frequency-sweeping and the ring-down approaches [28]. Closkey et al. proposed an experimental system identification method to estimate the orientation of the stiffness axes [29]. They applied modern system identification algorithms to determine the multi-variable input–output model based on the I/O data from the drive electrodes to the sense electrodes. From the model parameter matrices, the stiffness axes are estimated. Schein et al. proposed a parametric modeling technique to estimate the stiffness axes for degenerate modes from the response data [30]. In their approach, they overcame the difficulties confronted by frequency-response-based methods when the frequency split is too small. The modal frequencies, time constants, and stiffness axes can be determined simultaneously in their approach.

This paper provides theoretical analyses and detailed approaches to identify the stiffness axes based upon a measurement of phase differences in signals detected from two orthogonal sensing electrodes for different pattern angles. Firstly, in our approach, the theory is direct and simple; therefore, no time-consuming calculation is needed. Then, our approach shows a weak dependence on the resonant frequency, which means the method works well even when the natural frequency of the resonator has some significant variation due to environmental change. Moreover, our method works well for both large and small frequency splits. Most importantly, our approach focuses mainly on the characterization of the azimuth angle of the stiffness axes. The influences from the modal frequencies, damping, and damping asymmetries are mostly decoupled. Lastly, all external forces are switched off in the measurements; therefore, the errors from inaccurate forces or phases will be minimized.

The theoretical basis of our method is introduced in Section 2, followed by a detailed description of the procedures step by step. Section 3 validates our approach with a resonator gyroscope by comparing the experimental results with the simulations of the theoretical model. Section 4 concludes the paper with a summary of the results.

## 2. Materials and Methods

**Figure 1:**
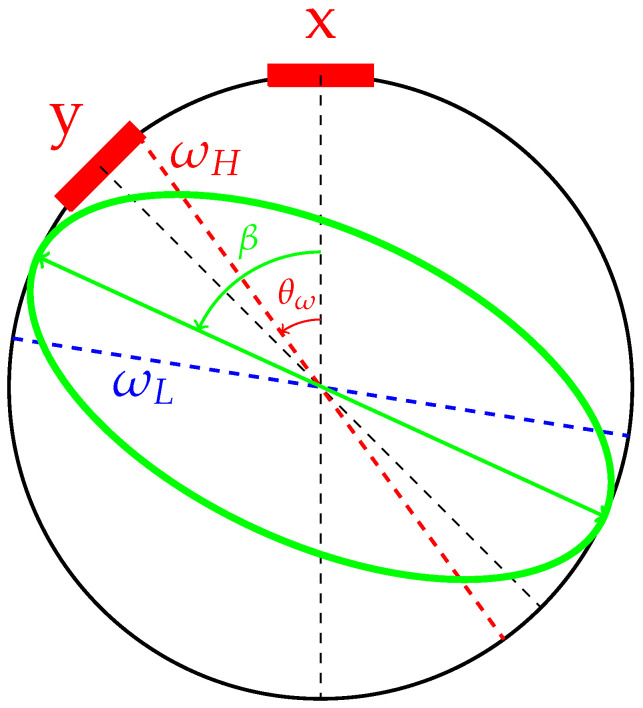
Schematic diagram of an imperfect axisymmetric resonator. Two red squares represent the orthogonal sensing electrodes. The dashed red line represents the high-frequency stiffness axis with an azimuth angle of $\theta_{\omega }$, and the dashed blue line corresponds to the low-frequency stiffness axis with an azimuth angle equal to $\theta_{\omega }+45^{{}^{\circ}}$. The green ellipse exhibits deformation of the resonator with the pattern angle β.

A typical axisymmetric resonator, operating in the n=2 mode of vibration, can be modeled as a two-dimensional harmonic oscillator. For the ease of illustration, we neglect damping mismatch, assume zero rotation input, and focus on effects of stiffness asymmetry on the dynamics of the system. The equations of motion can be expressed as [8,14]

(1) $$\begin{array}{c} \ddot{x}+\frac{2}{\tau }\dot{x}+\left(\omega^{2}+\omega \Delta \omega \cos4\theta_{\omega }\right)x+\omega \Delta \omega \sin4\theta_{\omega }y=\frac{f_{x}}{m}\\ \ddot{y}+\frac{2}{\tau }\dot{y}+\left(\omega^{2}-\omega \Delta \omega \cos4\theta_{\omega }\right)y+\omega \Delta \omega \sin4\theta_{\omega }x=\frac{f_{y}}{m} \end{array}$$

where *x* and *y* are displacements of the resonator detected by two orthogonal sensing electrodes. As depicted in Figure 1, the angle between two neighboring electrodes in our study is $45^{{}^{\circ}}$. The center frequency of the resonator is $\omega =\sqrt{\frac{\omega_{L}^{2}+\omega_{H}^{2}}{2}}$, with $\omega_{L}$ denoting the eigen frequency of the low-frequency axis and $\omega_{H}$ the eigen frequency of the high-frequency axis. The frequency split is defined by $\Delta \omega =\frac{\omega_{H}^{2}-\omega_{L}^{2}}{2\omega }$. The angle between the x signal and the high-frequency axis is denoted by $\theta_{\omega }$, which is the focus of the paper, as shown in Figure 1. The nominal energy decay time constant is denoted by τ. In this work, we ignore the damping asymmetry of the resonator, because the effect of damping on the dynamics of the resonator is trivial in our method when external control forces are turned off. The equivalent mass *m* is determined by the structure and material of the resonator. The external forces $f_{x}$ and $f_{y}$ are forces acting on x and y directions, respectively. The external forces are used for the energy compensation, the quadrature error elimination, and the pattern angle control.

To focus on the stiffness asymmetry of the system, we neglect the damping of the system and switch off all external forces temporarily. The equations of motion in this case can be reduced to

(2) $$\begin{array}{c} \ddot{x}+\left(\omega^{2}+\omega \Delta \omega \cos4\theta_{\omega }\right)x+\omega \Delta \omega \sin4\theta_{\omega }y=0\\ \ddot{y}+\left(\omega^{2}-\omega \Delta \omega \cos4\theta_{\omega }\right)y+\omega \Delta \omega \sin4\theta_{\omega }x=0 \end{array}$$

Furthermore, with the following orthogonal transformation

(3) $$\left(\begin{array}{c} x^{\prime }\\ y^{\prime } \end{array}\right)=\left(\begin{array}{cc} \cos2\theta_{\omega } & \sin2\theta_{\omega }\\ -\sin2\theta_{\omega } & \cos2\theta_{\omega } \end{array}\right)\left(\begin{array}{c} x\\ y \end{array}\right)$$

the equations can be easily simplified to

(4) $$\begin{array}{c} {\ddot{x}}^{\prime }+\left(\omega^{2}+\omega \Delta \omega \right)x^{\prime }={\ddot{x}}^{\prime }+\omega_{H}^{2}x^{\prime }=0\\ {\ddot{y}}^{\prime }+\left(\omega^{2}-\omega \Delta \omega \right)y^{\prime }={\ddot{y}}^{\prime }+\omega_{L}^{2}y^{\prime }=0 \end{array}$$

In the new representation, the two modes of vibration are fully decoupled. $x^{\prime }$ and $y^{\prime }$ describe two principal oscillations along two stiffness axes with natural frequencies of $\omega_{L}$ and $\omega_{H}$. From Equation (4), the motion of the resonator can be considered as a superposition of the two principal oscillations. The solutions of the two equations can be easily obtained as

(5) $$\begin{array}{c} x^{\prime }=A_{H}\cos\left(\omega_{H}t+\phi_{H}\right)\\ y^{\prime }=A_{L}\cos\left(\omega_{L}t+\phi_{L}\right) \end{array}$$

where the two oscillation amplitudes $A_{L}$ and $A_{H}$ and the two phases $\phi_{L}$ and $\phi_{H}$ are all completely determined by initial conditions of the resonator. From another perspective, we can set the values of $A_{L}$, $A_{H}$, $\phi_{L}$, and $\phi_{H}$ by controlling the resonator in different initial states.

Even though the analysis of $x^{\prime }$ and $y^{\prime }$ is simpler and more direct, the signals that can be directly extracted are from the two sensing electrodes *x* and *y*. The signals *x* and *y* can be calculated by the inverse transformation of (3), from $x^{\prime }$ and $y^{\prime }$.

(6) $$\begin{array}{cc} x & =\cos2\theta_{\omega }A_{H}\cos\left(\omega_{H}t+\phi_{H}\right)-\sin2\theta_{\omega }A_{L}\cos\left(\omega_{L}t+\phi_{L}\right)\\ y & =\sin2\theta_{\omega }A_{H}\cos\left(\omega_{H}t+\phi_{H}\right)+\cos2\theta_{\omega }A_{L}\cos\left(\omega_{L}t+\phi_{L}\right) \end{array}$$

Obviously, *x* and *y* are completely determined by directions of stiffness axes ($\theta_{\omega }$) and initial conditions ($A_{L}$, $A_{H}$, $\phi_{L}$, $\phi_{H}$) of the resonator. It is possible that by varying initial conditions and looking into the corresponding detected signals, the directions of stiffness axes can be obtained. The core idea in finding the stiffness axes is based on these facts:

- If the motion of the resonator has only one principal oscillation mode with nonzero amplitude, which means either $A_{L}$ or $A_{H}$ is zero, then the pattern angle will coincide with one of the two stiffness axes. As a result, the phase variations of the two detected signals will be synchronous, i.e., the two detected signals will have the same phase or the opposite phase, depending on the angles between the pattern angle and the directions of the x and y sensing electrodes.
- If both principal vibration modes have nonzero amplitudes, the pattern angle will be between the two stiffness axes. Then, the phase variations of the two detected signals will be asynchronous, i.e., the phase difference between *x* and *y* will vary with time.

To facilitate the phase comparison between the two detected signals, we firstly control the resonator in the force-to-rebalance (FTR) mode [31,32]: the two principal vibration modes are forced to oscillate at the same frequency and the phase difference between them is 0, i.e., $\phi_{L}-\phi_{H}=0$. In the FTR mode, the pattern angle can be controlled at an arbitrary direction by setting amplitudes $A_{L}$ and $A_{H}$ to some certain value. Secondly, we switch off all external forces, the detected signals will evolve as expressed in (6). By measuring the phase difference between *x* and *y*, we can tell whether the pattern vibration direction coincides with one of the two stiffness axes. The following paragraphs will give a more detailed mathematical description of our approach.

**Figure 2:**
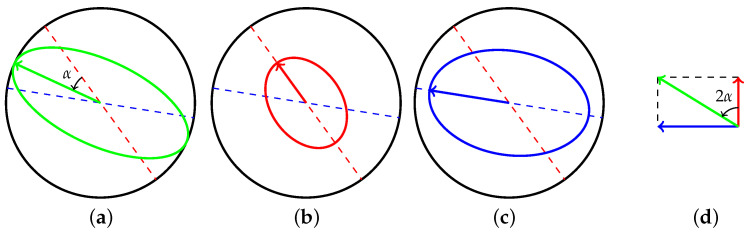
The vibration of the resonator in (**a**) can be decomposed as a vector superposition of vibration depicted in (**b**,**c**), with $\alpha =\beta -\theta_{\omega }$. The length of the arrows in these plots is a representation of the oscillation amplitudes. (**d**) is the corresponding ordinary Euclid orthogonal coordinate representation.

As shown in Figure 2, setting the resonator pattern angle to β, and defining angle $\alpha =\beta -\theta_{\omega }$, the motion of the resonator can be decomposed to two principal vibrations. The amplitudes of the two principal oscillations will be

(7) $$\begin{array}{cc} A_{H} & =A_{0}\cos2\alpha \\ A_{L} & =A_{0}\sin2\alpha \end{array}$$

The constant $A_{0}$, a measure of amplitude or energy of the whole pattern vibration, have little influence on the measurement results. However, it is worth noting that $A_{0}$ should not be too large so that the system is in the linear region [33].

At time t=0, we release the FTR control by switching off all external forces; as a result, the two principal oscillations recover their natural frequencies. The two detected signals then will evolve as

(8) $$\begin{array}{cc} S_{x} & =A_{0}\cos2\alpha \cos2\theta_{\omega }\cos\omega_{H}t-A_{0}\sin2\alpha \sin2\theta_{\omega }\cos\omega_{L}t\\ S_{y} & =A_{0}\cos2\alpha \sin2\theta_{\omega }\cos\omega_{H}t+A_{0}\sin2\alpha \cos2\theta_{\omega }\cos\omega_{L}t \end{array}$$

### 2.1. The Amplitudes of Detected Signals versus Time

Firstly, we study the amplitudes of the two detected signals as functions of time. The two detected signals (8) can be easily simplified to

(9) $$\begin{array}{cc} S_{x} & =A_{x}\cos\left(\omega_{H}t+\varphi \right)\\ S_{y} & =A_{y}\cos\left(\omega_{H}t+\varphi^{\prime }\right) \end{array}$$

with instantaneous amplitudes and phases defined by

(10) $$\begin{array}{cc} A_{x} & =\frac{A_{0}}{2}\sqrt{2+2\cos4\alpha \cos4\theta_{\omega }-2\sin4\alpha \sin4\theta_{\omega }\cos d\omega t}\\ A_{y} & =\frac{A_{0}}{2}\sqrt{2-2\cos4\alpha \cos4\theta_{\omega }+2\sin4\theta_{\omega }\sin4\alpha \cos d\omega t}\\ \varphi & =\arctan\frac{-\sin2\alpha \sin2\theta_{\omega }\sin d\omega t}{\cos2\alpha \cos2\theta_{\omega }-\sin2\alpha \sin2\theta_{\omega }\cos d\omega t}\\ \varphi^{\prime } & =\arctan\frac{\sin2\alpha \cos2\theta_{\omega }\sin d\omega t}{\cos2\alpha \sin2\theta_{\omega }+\sin2\alpha \cos2\theta_{\omega }\cos d\omega t} \end{array}$$

**Figure 3:**
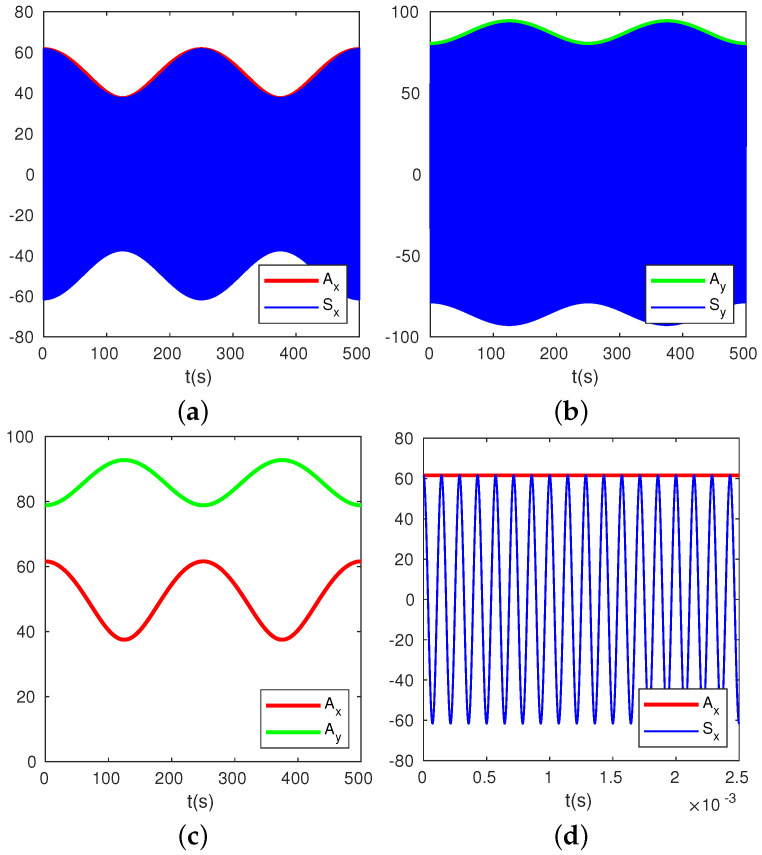
A numerical simulation of output signals $S_{x}$ and $S_{y}$ and amplitudes $A_{x}$ and $A_{y}$. Set $\theta_{\omega }=30^{{}^{\circ}}$, $\beta =26^{{}^{\circ}}$, f=7000 Hz, df=4 mHz. The thin blue lines show $S_{x}$ and $S_{y}$, and the thick red and green lines are for $A_{x}$ and $A_{y}$. Plot (**d**) is an amplification of plot (**a**) with a smaller time scale.

Without loss of generality, we set $\theta_{\omega }=30^{{}^{\circ}}$, $\beta =26^{{}^{\circ}}$, f=7000 Hz, df=4 mHz, and give a numerical simulation of $S_{x}$, $A_{x}$, $S_{y}$, and $A_{y}$ in Figure 3. Like most cases, both $A_{L}\neq 0$ and $A_{H}\neq 0$, the two detected signals $S_{x}$ and $S_{y}$ are additions of two waves with different frequencies, resulting in beats, as shown in Figure 3. From expression (10) and simulation results in Figure 3, it is obvious that the beat frequency is exactly the same to the frequency split $df=\frac{d\omega }{2\pi }$. The variations of $A_{x}$ and $A_{y}$ versus time are in opposite directions, i.e., when $A_{x}$ reaches the maximum, $A_{y}$ reaches the minimum, and vice versa.

**Figure 4:**
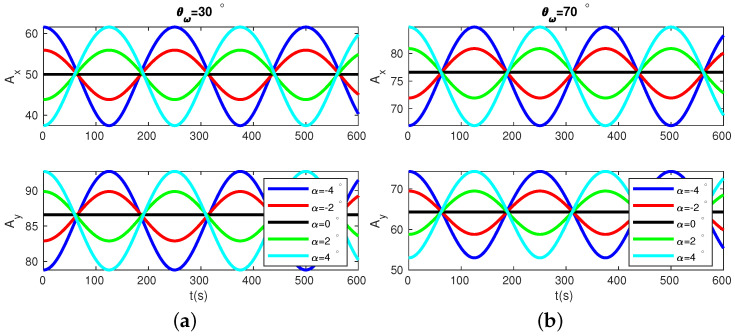
$A_{x}$ and $A_{y}$ as functions of time for different pattern vibration directions in two cases of $\theta_{\omega }$: $\theta_{\omega }=30^{{}^{\circ}}$ and $\theta_{\omega }=70^{{}^{\circ}}$. Black, green, cyan, red, and blue lines correspond to $\alpha =0^{{}^{\circ}}$, $2^{{}^{\circ}}$, $4^{{}^{\circ}}$, $-2^{{}^{\circ}}$, $-4^{{}^{\circ}}$, respectively.

A more detailed simulation of $A_{x}$ and $A_{y}$ with different pattern angles for two cases of $\theta_{\omega }$, $\theta_{\omega }=30^{{}^{\circ}}$ and $\theta_{\omega }=70^{{}^{\circ}}$, are plotted In Figure 4. In both cases, the variation ranges of $A_{x}$ and $A_{y}$ are closely related to α: the closer the pattern angle is to the stiffness axes, the smaller variation range $A_{x}$ and $A_{y}$ will be; when the pattern angle coincides with either of the stiffness axes, $A_{x}$ and $A_{y}$ will be constant. In theory, we can use this phenomenon to search for the stiffness axes of the resonator. However, the resolution of this approach by observing signal amplitudes variation range is limited in experiments. When the pattern angle is close to the stiffness axes, the amplitude variation range is so small that it is difficult to tell whether the amplitude variation range is increasing or decreasing with small variations of pattern angles.

**Figure 5:**
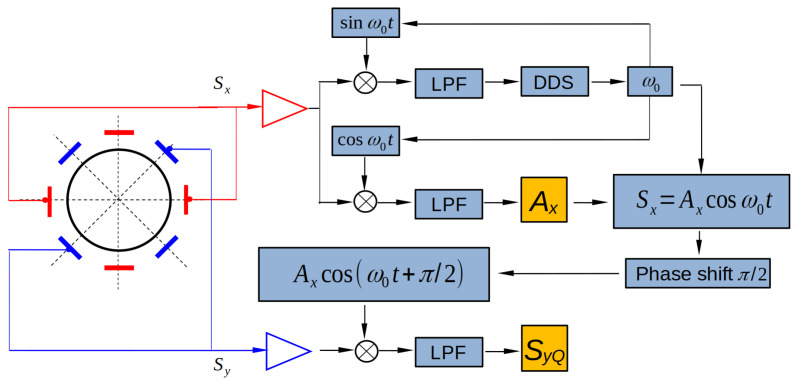
Details of signal processing to obtain the amplitudes $A_{x}$ and the demodulated signal $S_{yQ}$.

Experimentally, the amplitudes $A_{x}$ can be obtained easily from detected signal $S_{x}$ by means of conventional phase-locked loop (PLL), as shown in Figure 5.

### 2.2. The Demodulated Signal versus Time

The main information we utilized to identify the azimuth angles of the two stiffness axes is the phase difference between the detected signals from x and y channels, which can be analyzed through demodulation, as described in Figure 5. Using the PLL, we trace the phase and amplitude of the detected signal $S_{x}$, and then the phase is shifted for $\frac{\pi }{2}$. This shifted signal is then multiplied by the other detected signal $S_{y}$, and then passed through a low-pass filter. Theoretically, the final demodulated signal, denoted by $S_{yQ}$, can be calculated as

(11) $$\begin{array}{c} S_{yQ}=\frac{1}{4}A_{0}^{2}\sin4\alpha \sin d\omega t \end{array}$$

When the resonator is controlled in the FTR mode, the two principal vibration modes are forced to oscillate at the same frequency, i.e., dω=0, and the demodulated signal $S_{yQ}$ will remain zero. When all the control forces are switched off, the demodulated signal $S_{yQ}$ will evolve as expressed by Equation (11). It is worth noting that Equation (11) shows no direct dependence on $\theta_{\omega }$ or β but only on their difference α. The slope of $S_{yQ}$ over time is used to tell whether the current β coincides with either stiffness axes

(12) $$\begin{array}{c} {\dot{S}}_{yQ}=\frac{1}{4}A_{0}^{2}\sin4\alpha \;d\omega \cos d\omega t \end{array}$$

**Figure 6:**
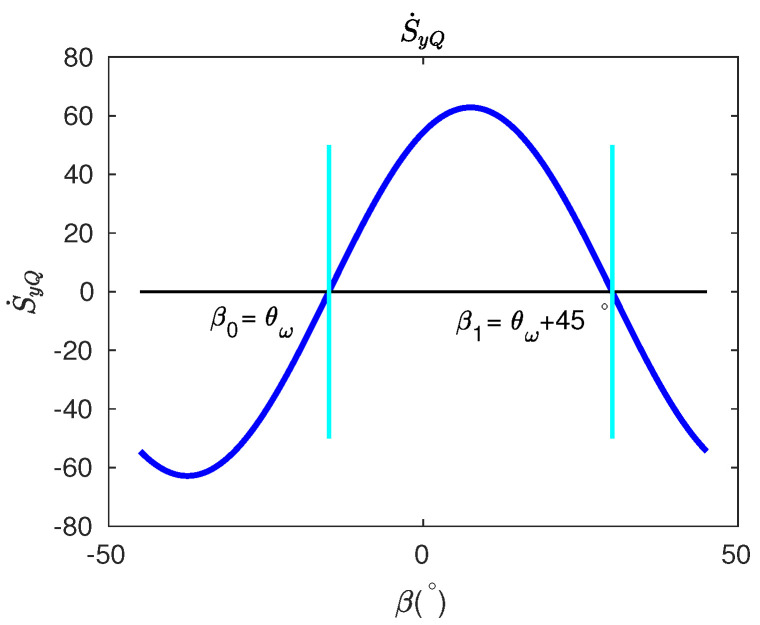
The demodulated quantity ${\dot{S}}_{yQ}$ as function of pattern angle β, with $\theta_{\omega }$ set to $-15^{{}^{\circ}}$.

The dependence of ${\dot{S}}_{yQ}$ on β, in the range of $-45^{{}^{\circ}}$ to $45^{{}^{\circ}}$, is plotted in Figure 6 with $\theta_{\omega }=-15^{{}^{\circ}}$. At two critical pattern angles when $\beta_{0}=\theta_{\omega }$ and $\beta_{1}=\theta_{\omega }+45^{{}^{\circ}}$, we have ${\dot{S}}_{yQ}=0$. At the high-frequency axis when $\beta =\beta_{0}$, the slope of ${\dot{S}}_{yQ}$ over β is positive. At the low-frequency axis $\beta =\beta_{1}$, however, the slope of ${\dot{S}}_{yQ}$ over β is negative. In short, when the pattern angle coincides with either stiffness axes, ${\dot{S}}_{yQ}$ will be zero, and we can distinguish whether the stiffness axis is the low-frequency or the high-frequency axis by observing the slopes of ${\dot{S}}_{yQ}$ over β at two ${\dot{S}}_{yQ}=0$ angles.

A detailed procedure for the identification of stiffness axes can be summarized as follows: First, the resonator is controlled in FTR mode with pattern angles β varying from $-45^{{}^{\circ}}$ to $45^{{}^{\circ}}$ with a step of appropriate angles. At different βs, after the FTR control is stabilized, all control forces are switched off and then the development of the demodulation quantity $S_{yQ}$ is observed for a few seconds; two specific βs, where ${\dot{S}}_{yQ}=0$, correspond to the two azimuth angles of the two stiffness axes. We can tell either β corresponds to the low-frequency or the high-frequency stiffness axis by measuring slopes of ${\dot{S}}_{yQ}$ over β, for which past studies are not discussed in detail.

Even though the damping of the resonator does not have evident influence on the measurement results in this study, it exists in real measurement. For the purpose of comparing with experimental results, an exponential decay of amplitudes is included in the simulations in the following studies.

## 3. Results and Discussion

**Figure 7:**
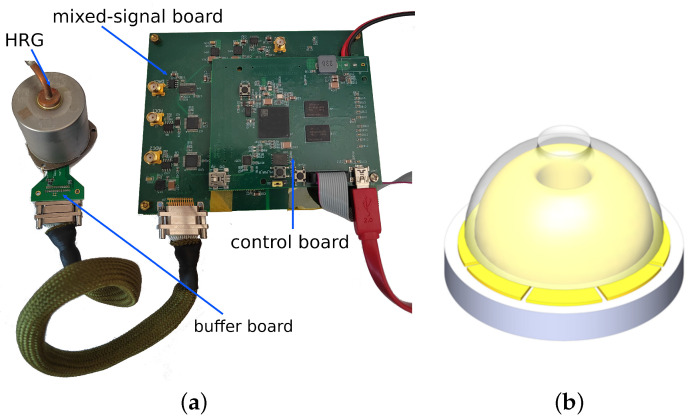
(**a**) Photograph of the experimental setup with an HRG and a circuits system. (**b**) Physical structure of the HRG.

**Figure 8:**
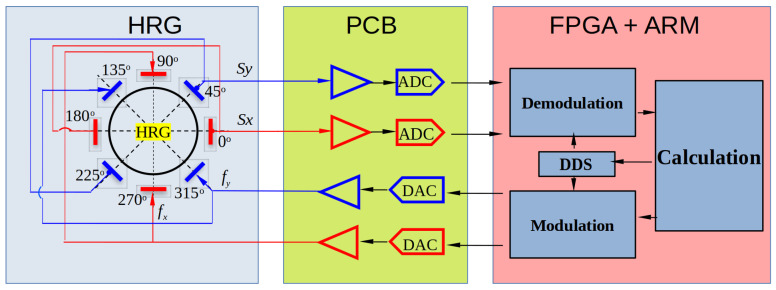
Block diagram of the HRG with the circuits system. The PCB part shows a sketch of functions for both the buffer board and the mixed-signal board. The FPGA + ARM part corresponds to the digital control board.

To verify the theoretical analyses and confirm the validity of our method, experiments are conducted for comparison and verification. The experimental setup is displayed in Figure 7a, including a prototype of a hemispherical resonator gyroscope (HRG) and a circuits system. The HRG prototype is composed of a fused silica hemispherical resonator with a high-quality factor and an electrode plate with eight discrete electrodes, as shown in Figure 7b. The electrical connection and control strategy of the circuits system are depicted in Figure 8. The applications of the eight electrodes are clearly shown. The electrodes separated by 180 degrees are connected directly. The x sensing direction is defined as the $0^{{}^{\circ}}$ direction and the y sensing direction as $45^{{}^{\circ}}$. The electrodes with directions orthogonal to the sensing electrodes directions are used for driving. The circuits system consists of a buffer board mounted on the HRG, a mixed-signal board with two analog-to-digital converters (ADC) and two digital-to-analog converters (DAC), and a digital control board, mounted on the top of the mixed-signal board, with both FPGA and ARM functions. The buffer board converts the detected current signals to the appropriate voltage signals, then the mixed-signal board converts the analog voltages to digital signals with two ADCs and generates drive forces with two DACs. The control board implements the force-to-rebalance (FTR) control and the measurement algorithms. The FPGA part performs high-speed demodulation, modulation and DDS processes, and the ARM part performs the other low-speed but more complicated FTR or measurements functions.

Utilizing the proposed procedure, we measured the natural frequencies and the stiffness axes of the resonator, as listed in Table 1. The resolution of the measured stiffness axes angles is better than $0.1^{{}^{\circ}}$, which means if we change the β by $0.1^{{}^{\circ}}$ around each stiffness axis, we can see the opposite sign of ${\dot{S}}_{yQ}$. It should be noticed that the angle between the two stiffness axes obtained is $\beta_{1}-\beta_{0}=43.3^{{}^{\circ}}<45^{{}^{\circ}}$, which is not consistent with the theoretical expectation. This discrepancy could probably be attributed to the fact that the two sensing electrodes are not exactly orthogonal to each other: the gains of the two sensing channels are not the same, or the angle between the two sensing electrodes is not exactly $45^{{}^{\circ}}$. This leads to a small deviation in all the measured angles. In this work, we neglect this effect for the moment, and our future work will focus on this problem.

To check the validity of our method, we plotted the amplitudes $A_{x}$ and demodulated signals $S_{yQ}$ as functions of time around the stiffness axes directions based on the experimental measurements and compared the experimental results with the theoretical predictions. For a clear illustration, we use the same parameters as listed in Table 1 for the theoretical simulations, except for a change of $\beta_{1}$ to $\beta_{1}=\beta_{0}+45^{{}^{\circ}}$ instead of $\beta_{1}=\beta_{0}+43.3^{{}^{\circ}}$ so as to be consistent with the theory.

### 3.1. Amplitudes versus Time

**Figure 9:**
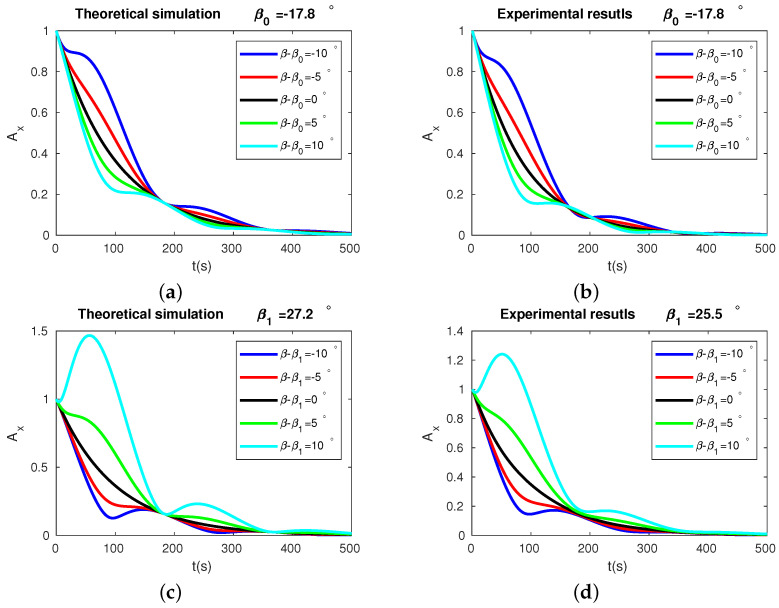
Results of theoretical simulations and experiments of $A_{x}$. In plot (**a**,**b**), the black, green, cyan, red, and blue lines correspond to $\beta -\beta_{0}=0^{{}^{\circ}}$, $5^{{}^{\circ}}$, $10^{{}^{\circ}}$, $-5^{{}^{\circ}}$, $-10^{{}^{\circ}}$, respectively. In plot (**c**,**d**), the black, green, cyan, red, and blue lines correspond to $\beta -\beta_{1}=0^{{}^{\circ}}$, $5^{{}^{\circ}}$, $10^{{}^{\circ}}$, $-5^{{}^{\circ}}$, $-10^{{}^{\circ}}$, respectively.

The amplitude behavior as the function of time of the resonator is presented in Figure 9. Subfigures (a) and (c) are the numerical simulations, while subfigures (b) and (d) are the experimental results. Subfigures (a) and (b) are the amplitude behavior of the detected signals for the pattern angles near $\beta_{0}=-17.8^{{}^{\circ}}$, while subfigures (c) and (d) are for the pattern angles near $\beta_{1}$. The different colors represent different βs. From the two figures, it is clearly shown that, except for the exponential decay from damping, the closer the pattern angle to the stiffness axes angle, the smaller the vibration amplitude fluctuations will be. The experimental results agree well with the numerical simulations, which partly proved the validity of our method.

### 3.2. Demodulated Signals versus Time

**Figure 10:**
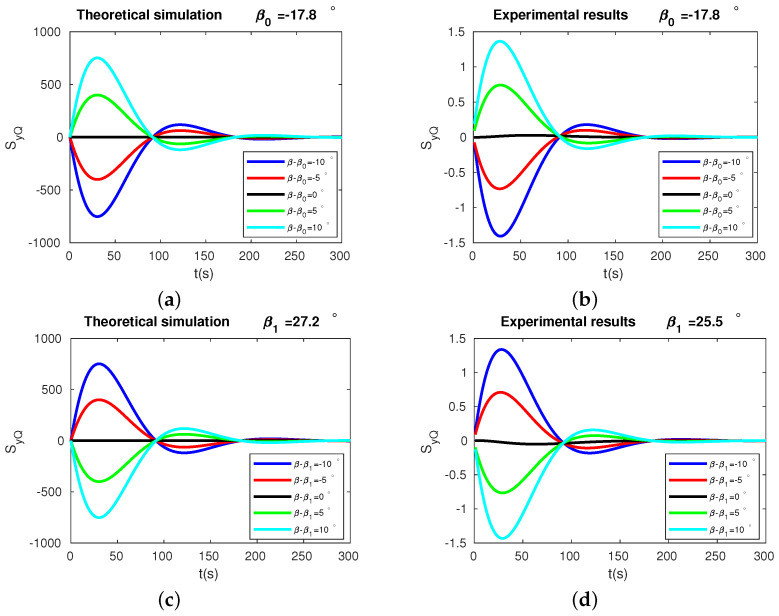
Theoretical results and experimental results of $S_{yQ}$. In plot (**a**,**b**), the black, green, cyan, red, and blue lines correspond to $\beta -\beta_{0}=0^{{}^{\circ}}$, $5^{{}^{\circ}}$, $10^{{}^{\circ}}$, $-5^{{}^{\circ}}$, $-10^{{}^{\circ}}$, respectively. In plot (**c**,**d**), the black, green, cyan, red, and blue lines correspond to $\beta -\beta_{1}=0^{{}^{\circ}}$, $5^{{}^{\circ}}$, $10^{{}^{\circ}}$, $-5^{{}^{\circ}}$, $-10^{{}^{\circ}}$, respectively.

The demodulated signal $S_{yQ}$ as the function of time, from both the experimental measurements ((b) and (d)) and theoretical predictions ((a) and (c)), for different pattern angles, are shown in Figure 10. The different colors represent different βs. Subfigures (a) and (b) present the results for the pattern angles near the high-frequency stiffness axis, $\beta_{0}=-17.8^{{}^{\circ}}$. When $\beta <\beta_{0}$, we see a negative slope of the demodulated signal $S_{yQ}$ dependence on time in the first few seconds. When $\beta >\beta_{0}$, the sign of the slope ${\dot{S}}_{yQ}$ switched from negative to positive. Subfigures (c) and (d) show the behavior of the demodulated signal around the low-frequency stiffness axis. In contrast to the case with the high-frequency stiffness axis, we see a positive slope of $S_{yQ}$ at pattern angles smaller than $\beta_{1}$ and a negative slope at pattern angles larger than $\beta_{1}$. Again, we see the simulation results fit well with the experiments. These results confirmed the validity of our method from a different perspective.

## 4. Conclusions

In conclusion, this paper introduced a novel high-precision identification method of stiffness axes for axisymmetric resonators. Ignoring both the effects of damping asymmetry and the external rotation, the whole motion of the resonator can be decomposed as a superposition of two independent principal oscillations with two different natural frequencies along two stiffness axes. This method is based on comparing the phase differences in the two detected signals. The method was applied on a resonator gyroscope with an electrode plate of eight independent electrodes, and the stiffness axes angles measured show a resolution better than $0.1^{{}^{\circ}}$. Finally, a comparison of the detected signal amplitudes $A_{x}$ and demodulated signals $S_{yQ}$ for different pattern angles between the experimental results and theoretical prediction were carried out. A good agreement between them confirmed the validity of the proposed method.

Lastly, even though we take electrostatic sensing and actuation in our experiments on an HRG prototype in this paper, our method is not restricted to these types of actuation or sensing mechanisms and is not limited to HRGs. The core idea behind our method is that the resonator can be excited in any selected pattern angles and can be detected from two non-parallel or non-identical directions. When the resonator is excited in a certain pattern angle, and no clear phase difference deviation is observed in a short time after all the forces are released, the certain angle corresponds to one of the stiffness axes angles.

## Figures and Tables

**Table 1:** Experimental results and measured parameters of the HRG; $\beta_{0}$ and $\beta_{1}$ represent directions of high-frequency stiffness axis and low-frequency stiffness axis, respectively.

Real Parameters	Value	Units
center frequency	6427	Hz
frequency split	5.47	mHz
τ	100	s
$\beta_{0}$	−17.8	${}^{{}^{\circ}}$
$\beta_{1}$	25.5	${}^{{}^{\circ}}$

## Data Availability

The data that support the findings of this study are available from the corresponding author upon reasonable request.

## Abbreviations

The following abbreviations are used in this manuscript:

CVG	Coriolis vibratory gyroscopes
FTR	Force to rebalance
PLL	Phase-locked loop
